# Life-long phishing attack detection using continual learning

**DOI:** 10.1038/s41598-023-37552-9

**Published:** 2023-07-17

**Authors:** Asif Ejaz, Adnan Noor Mian, Sanaullah Manzoor

**Affiliations:** Department of Computer Science, Information Technology University, Lahore, 54000 Pakistan

**Keywords:** Mathematics and computing, Computer science

## Abstract

Phishing is an identity theft that employs social engineering methods to get confidential data from unwary users. A phisher frequently attempts to trick the victim into clicking a URL that leads to a malicious website. Many phishing attack victims lose their credentials and digital assets daily. This study demonstrates how the performance of traditional machine learning (ML)-based phishing detection models deteriorates over time. This failure is due to drastic changes in feature distributions caused by new phishing techniques and technological evolution over time. This paper explores continual learning (CL) techniques for sustained phishing detection performance over time. To demonstrate this behavior, we collect phishing and benign samples for three consecutive years from 2018 to 2020 and divide them into six datasets to evaluate traditional ML and proposed CL algorithms. We train a vanilla neural network (VNN) model in the CL fashion using deep feature embedding of HTML contents. We compare the proposed CL algorithms with the VNN model trained from scratch and with transfer learning (TL). We show that CL algorithms maintain accuracy over time with a tolerable deterioration of 2.45%. In contrast, VNN and TL-based models’ performance deteriorates by over 20.65% and 8%, respectively.

## Introduction

Phishing is a technique in which a cyber-criminal (also known as an attacker) clones a website’s interface and sends a compelling message to a naive user through an email or social media chat to open the link in that message. A similar but cloned interface is opened when a user opens the link. Any username and password entered in this interface is sent to the attacker, which can then be exploited. The number of phishing attacks is increasing. According to the anti-phishing working group (APWG), 316, 747 phishing attacks were reported in December 2021, which was the highest monthly total in APWG’s history^1^.

Phishing attacks have become a serious threat and need to be detected on the fly before the user gets trapped. Over the years, phishing attacks have matured by using advanced phishing methods and web development technology to become less prone to detection^2^, and they are continuously evolving and adapting to evade current intrusion detection systems and intrusion prevention systems. Generally, phishing detection systems can be classified into two groups, i.e., rule-based and ML-based^3^. Rule-based phishing detection systems blacklist malicious domains and URLs^4^. A manual effort is required to update the new domains and websites. Such systems cannot detect a first-time attack (zero-day attack). Also, these cannot detect false positive incidents (samples that are normal, but the system has predicted them as malicious) due to human error in labeling^2^. ML-based techniques, on the other hand, utilize the historical data of phishing pages to find patterns in the webpage content^5–8^ and URLs^4,9,10^ of web pages. ML-based methods are now state-of-the-art in phishing detection as they perform better than rule-based detection systems^4,11,12^. Although ML-based methods are effective in phishing detection, these systems, because of being trained on historical datasets, fail to detect sophisticated crafted phishing samples in the future due to changes in feature distribution. Regular model retraining or TL on these upcoming samples mitigates this issue but leads to performance deterioration in the older samples detection. To tackle this problem, we adopted CL-based algorithms to maintain the performance of old and new phishing attacks detection. In TL, the last few layers of an existing model are retrained to adapt it to a new dataset, while in CL, a model is retrained to adapt to new data while maintaining performance on previous data. We shall discuss details of these methods in Section “Methodology”.

The specific contributions of this study are: (i) we identify performance deterioration over time in traditional ML systems for phishing detection due to excessive changes in feature distribution and the evolution of web development technologies, (ii) we propose a CL-based phishing detection framework to cope with performance drop issues in traditional ML models and show that the approach improves learning performance with limited training data and requires less retraining time.

The rest of the paper is organized as follows. We provide related work in Section “Related Work”. We define research tools and methods in Section “Research tools and methods”. We present our methodology in Section “Methodology”. We describe experiments and results in Section “Performance comparison of continual learning”. We perform analysis in Section “Discussions”. Finally, we conclude in Section “Conclusion and future work”.

## Related Work

As phishing attacks grow, researchers are putting efforts into providing reliable and resilient solutions for automatically detecting phishing attacks. Current ML-based phishing detection techniques are classified based on features used for detection, i.e., URL features, content features, and visual and hybrid features based detection. These detection techniques are discussed in the following section.

### URL features based detection

Phishing detection using URLs is effective for offline training data. Few studies^4,7,13^ have identified and extracted hand-crafted features from URLs and used these features all alone or in combination with content-based features to train ML models. They used support vector machine (SVM), decision tree (DT), and random forest (RF) models and achieved more than 95% accuracy. Recent studies^14–18^ have used deep learning-based methods such as convolutional neural networks (CNN) and generative adversarial networks (GAN). URLs are generated through the GAN model to solve the data bias problem caused by the imbalance in phishing datasets. Their method achieved an accuracy of 95.6%. Wei et al.^15^ proposed a novel multi-spatial character-level model that is applied to URLs using (CNN) for fast and accurate phishing detection. Patil et al.^16^ reviewed URL-based techniques and developed a model to overcome the issues of bias found in previous works. Sherazi et al.^10^ observed that URL is not the best way to detect a phishing website. Their proposed system uses only domain names, as phishers can control the URL but can not change the domain name. They developed a faster and unbiased system with only seven features and an accuracy of 97-99.7%. Tian et al.^2^ highlights that URL-based detection methods have some limitations as particular domain names, such as internationalized domain names, allow attackers to register domains similar to some famous domains using different characters from local language to look similar to a legitimate domain. Furthermore, all of these URL-based detection methods lack the detection of advanced phishing techniques because they are not using webpage content features.

### Content based detection

Recent studies used particular keywords from webpage content as discriminative features for more robust phishing detection. Some researchers have employed ML algorithms to tackle the phishing detection problem and achieved promising results^6,8,11,19–24^. Advancements in both feature extraction and detection models have been made in these studies, for instance, content-based features extracted with NLP and reinforcement learning, ensembles and bagging for training, and others. Many recent studies used NLP-based feature extraction on email manuscripts and trained the ML models^6,19,23,25^. These studies proved the success of the NLP-based features in detecting phishing emails with an accuracy of 98.2%. Smadi et al.^11^ used reinforcement learning and extracted four features, including embedded URLs, HTML content, email header, and email manuscript features. With a dataset of approximately ten thousand data samples, they obtained 98.6% detection accuracy. Ubing et al.^20^ used RF to extract features, and the nine best content-based features out of 30 were selected and were used to train ensemble classifiers. Their ML system used a majority voting method to avoid model overfitting and achieved 95% accuracy.

Zamir et al.^21^ introduced a new approach using multiple ML algorithms. They used an ensemble of RF, neural networks (NN), and Bagging to achieve 97.4% accuracy in detecting phishing web pages. Their study shows that ensemble techniques are among the best detection strategies for phishing web pages detection. Niakanlahiji et al.^22^ crafted many features from HTML content, code complexity, and certificate features to devise a target-independent detection method. As a result, they achieved 95.4% accuracy with RF. Zheng et al.^26^ uses feature embedding and NN to detect phishing pages. It also combines character-level information with word-level information while embedding features. Their results are quite promising, with an accuracy of 98.30%, a true positive rate of 99.18%, and a true negative rate of 94.34%.

Liu et al.^27^ designed a multistage model that applies initial filters to detect phishing pages, such as the number of page views, etc. Moreover, they proposed a framework called CASE for extensive feature extraction. Their proposed multistage model with the CASE framework could achieve a recall of 0.9436, a precision of 0.9892, and an F1 measure of 0.9659. Tan et al.^28^ proposed a new technique of detection based on graph theory. They used the hyperlinks on the page to create the graph features to represent deeper information between features. Their experimental results showed an accuracy of 97.8%. Yi et al.^24^ presented three types of web phishing detection features: original and interaction features. It then used deep belief networks. This model achieved around 90% true positive rate and 0.6% false positive rate.

Some studies used feature selection methods to improve accuracy. For instance, Chiew et al.^29^ proposed a new feature selection technique called the hybrid ensemble feature selection. It consists of two phases that produce a set of baseline features. The hybrid ensemble feature selection shows the best results when used with the RF classifier, which achieves an accuracy of 94.6% with only 20.8% of the features used originally.

All content-based techniques rely on content-specific features. These techniques are robust to some extent but can also not capture advanced evasion techniques. When attackers use code obfuscation, then hand-crafted, content-based features will not be helpful anymore for phishing page detection. Furthermore, these techniques require time to process website content during runtime. So, there is a need for fast and efficient methods that use a deep vector embedding representation of content and are resilient to phishing web pages detection.

### Visual and hybrid features based detection

Some recent works used visual-based features combined with text features to improve phishing detection. Visual features are computed with the cosine similarity of the phishing page with the corresponding legitimate page^8,12,17,30–32^. Rao et al.^12^ have used a hybrid detection approach that employs both ML-based and visual similarity-based detection. This detection mechanism requires a vast image database to compare phishing web pages. They achieved 99.55% accuracy using HTML content-based features, URL-based features, and some third-party features. However, third-party features (online features) are costly and slow, making this approach impractical for real-time deployment.

Tial et al.^2^ identified squatting domain classes, obfuscation techniques, and essential features for robust learning. They extracted keywords from visual screenshots of pages using an optical character recognizer (OCR). They also converted code to vectors using feature embeddings to develop a robust detection model. They achieved 97% accuracy with RF, which is better than Naive Bayes and KNN. On the other hand, Chiew et al.^29^ used the website’s logo to detect phishing websites by applying a two-stage method. The first stage is extracting the logo from the website and using Google image search to find the domain name corresponding to the extracted logo. The method then compares the domain from the query website with the domain retrieved and classifies them according to URLs.

The visual and hybrid features-based techniques are pretty robust in phishing detection. However, these techniques pose a new challenge to the computational complexity of the model and the time to extract all features on runtime. Moreover, the existing literature on phishing detection has yet to consider the performance deterioration in ML-based systems over time. Thus, we need an efficient, robust, and adaptive model that solves existing work problems.

To the best of our knowledge, prior research has exclusively employed traditional VNNs, which have limitations in addressing novel phishing techniques until they are trained or fine-tuned on newly acquired data. Also, these methods may face performance drops when they are deployed in scenarios where phishing attacks are changing rapidly. Therefore, life-long phishing detectors are useful to deploy in those scenarios where retaining all previous data is costly and phishing attacks are evolving continuously. Also, they can be deployed to detect zero-day attacks. Also, CL algorithms are generally used in computer vision to retrain existing models to adopt new tasks without forgetting knowledge of prior tasks. We have applied CL algorithms like learning without forgetting (LWF)^33^ and elastic weight consolidation (EWC)^34^ to reduce the systematic performance drop in phishing detection models. LWF is a technique that enables an ML model to learn new tasks without forgetting previously learned knowledge. EWC is a method that selectively reinforces the important weights of a neural network to prevent catastrophic forgetting.

## Research tools and methods

In this section, we briefly describe different embedding methods and TL techniques.

### Embedding Techniques in ML

There is a range of NLP embedding models with different sizes and capabilities. The specific task requirements and computation resource availability can influence the selection of an embedding model. Some well-known embedding models are Word2Vec, Glove, FastText, ELMo, and BERT, which are briefly described as follows.

Mikolov et al.^35^ proposed a model called Word2Vec, which is trained on Wikipedia pages, and it effectively learns the semantic relationships between words. It has two variants: a continuous bag of words (CBOW) and a skip-gram. The CBOW model predicts a target word based on its context, while the Skip-gram model predicts the context words based on a target word. Word2Vec embeddings have sizes in the range of 100–300.

Pennington et al.^36^ introduced global vectors for word representation (GloVe). The gloVe is another widely used word embedding technique that is trained on large co-occurrence matrices of words to generate embeddings that capture both syntactic and semantic relationships between words. GloVe embeddings are of sizes from 100 to 300 and are known for their effectiveness in capturing global word co-occurrence patterns. However, they may not perform well on out-of-vocabulary words.

Bojanowski et al.^37^ presented a method called FastText, which is based on the Word2Vec model, but it also incorporates subword information. It breaks down each word into smaller character-based n-grams and learns embeddings for each n-gram with the complete word. This allows FastText to capture morphological information and handle rare and out-of-vocabulary words better than other embedding techniques. FastText embeddings are typical of sizes in the range of 100–300.

Devlin et al.^38^ designed bidirectional encoder representations from transformers (BERT), a state-of-the-art word embedding technique that uses a transformer model to generate contextualized word embeddings. It is trained on large amounts of text data in an unsupervised manner to generate embeddings that capture the meaning of words in context. BERT embeddings are typically of size in the range of 768–1024, and they are known for their effectiveness in learning complex NLP tasks such as question-answering, etc.

Cer et al.^39^ suggested a method called embeddings from language models (ELMo), which is a contextualized word embedding technique that generates embeddings based on the entire sentence context. It uses a bidirectional LSTM neural network to generate embeddings that capture the meaning of words in context. ELMo embedding vector is 1024 dimensional, and they are known for their effectiveness in capturing complex relationships between words and handling polysemy. However, they can be computationally expensive to train and require a large amount of training data.

After studying these embedding techniques from the literature, we found FastText suitable as it is a good balance between embedding size and vector quality, making it a popular choice for many NLP tasks. Also, FastText is trained on a common crawl dataset consisting of web pages related to our problem. Word2Vec and Glove are small and used for non-complex tasks, while ELMo and BERT are very complex models used for complex tasks.

### TL Techniques

TL is an ML technique that involves taking knowledge learned from one task and applying it to a new problem. A few TL-based techniques are; (i) fine-tuning, (ii) CL, (iii) domain adaptation (DA), (iv) progressive neural networks (PNNs), and (v) multi-task learning. These techniques are briefly explained as follows.

Fine-tuning involves taking a pre-trained model and adjusting it to perform a new task. The pre-trained model is usually trained on a large dataset, and the fine-tuning process involves training the model on a smaller, task-specific dataset. During fine-tuning, the weights of the pre-trained model are adjusted to better fit the new dataset while retaining the learned features from the original dataset. This process can significantly reduce the training time and computational resources required to train a new model from scratch^40^.

Lange et al.^41^ proposed a method called CL that adapts to new information over time. The main objective of CL is to develop models to learn and retain new information without forgetting the previously learned knowledge. CL is critical in real-world applications where data is constantly changing or evolving. Various CL algorithms, such as regularization, LWF, EWC, etc., are used to improve the ML models continuously over time.

In multi-task learning, multiple tasks are learned in parallel to generalize the ML model. So, it requires data availability for all tasks to be present before training. While in life-long learning scenarios, the model can be adapted for a new task at any time using only new available data^42^.

Rusu et al.^43^ introduced PNNs, which consist of a series of neural networks, each specialized in performing a specific task. Each network is trained on its specific task and can be used independently to make predictions. When a new task is introduced, a new network is added to the series, but the previously learned networks are frozen and kept unchanged. PNNs require a careful design of the network architecture and the training procedure to ensure that the added columns of neurons do not interfere with the existing knowledge. PNN is challenging to implement and optimize because it is computationally expensive, hence, infeasible for life-long learning.

In DA, an ML model which is previously trained on the source domain is adapted to the target domain, where the output is the same for the source and target domain, but the input data distribution is entirely different, requiring data availability for both domains that increase training complexity, high storage, and computation cost, and may face catastrophic forgetting as the number of retraining iterations increases, hence, infeasible for life long learning^44^.

After studying different TL techniques, we selected the fine-tuning and CL-based algorithms as they require less retraining time and have low computation costs.

## Methodology

**Figure 1:**
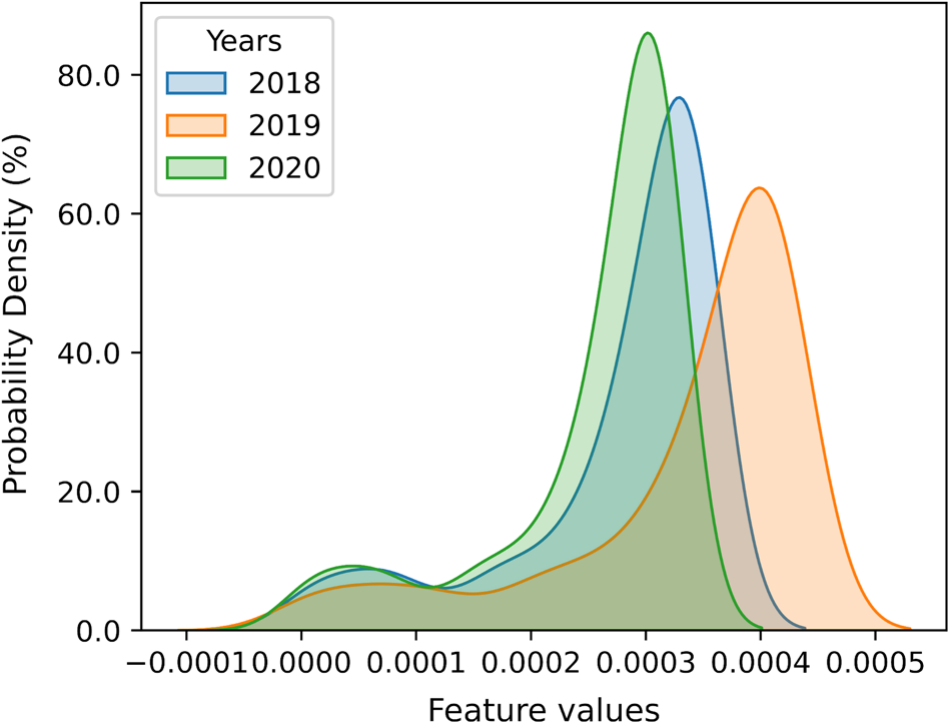
Distributions shift in one-dimensional PCA representation for three years data.

**Figure 2:**
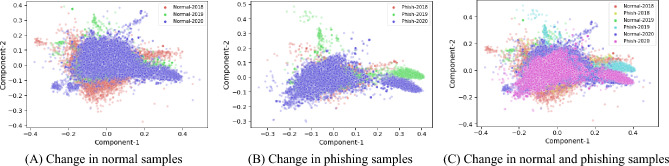
Distributions shift in two-dimensional PCA representation for three years data.

This section covers a detailed discussion of our methodology to identify and mitigate the performance drop. We analyzed our dataset features using low-dimensional principal component analysis (PCA) embedding of samples to visualize the difference in distribution. We transformed features into 1-D and 2-D PCA as shown in Figs. 1 and 2, respectively. Figure 1 shows the features distribution of 1-D PCA trends for data samples taken from 3 consecutive years, and the x-axis represents 1-D feature values while the y-axis represents the frequency of samples. It depicts the change in the distribution of features with one year gap. Similarly, we analyzed 2-D PCA features in Fig. 2. Figure 2a shows the scatter graph for all normal samples. Figure 2b shows the distribution of all phishing samples, and similarly, Fig. 2c shows the distribution for all phishing and normal samples collected in 3 consecutive years. These results show that feature distribution shift over the years is inevitable, which implies that one model trained on historical data can not perform consistently well in subsequent years’ data. Therefore, a solution is required to cope with this changing data distribution.

### Dataset

To the best of our knowledge, there is no standard dataset for phishing samples. We, therefore, collected phishing samples that are reported on community platforms (like VirusTotal^45^ and PhishTank^46^). The dataset contains extensive samples for training ML models across subsequent years to understand the performance drop over time. The dataset contains two types of web pages, i.e., phishing and benign. Their details are as follows.

- *Phishing samples *This set contains around 90k HTML web page samples that are submitted on famous community websites (PhishTank and VirusTotal) as malicious pages. This dataset contains only those samples which are active during the consecutive three years, i.e., 2018–2020, and marked as phishing samples by more than ten reputed Antivirus vendors such as VirusTotal.
- *Benign samples* This dataset contains around 80k HTML web pages submitted on VirusTotal in the same three years and not marked as phishing or malicious by more than 3 Antivirus vendors. We use a low threshold for Antivirus (AV) detection because some AV vendors are known to be highly sensitive, and they sometimes detect unknown or non-famous benign websites as phishing. Due to this high sensitivity of phishing detection of AV vendors, any web page with three or fewer phishing detections is considered a benign sample. Table 1 shows the number of samples per year.

**Table 1:** Dataset distribution.

Years	Phishing	Benign		
2018	17,854	16,814		
2019	20,538	11,605		
2020	61,112	52,663		

We performed training and testing experiments with six datasets to show the performance drop. We divided the three consecutive years (from 2018 to 2020) datasets into two equal sizes. Datasets are named as 2018A, 2018B, 2019A, 2019B, 2020A and 2020B.

### Data pre-processing and features extraction

First, as a pre-processing step, we excluded anomalous website samples, such as pages with very little HTML content. In the next step, we analyzed dataset features for ML model training. Many hand-crafted features proposed in recent studies are helpful in phishing detection with ML^13,20,21^. These features must be more exhaustive to detect advanced phishing attacks as websites evolve continuously. We employ deep feature representation of full HTML content (code and text) to address this issue using vector embedding. Vector embedding contains compressed and important information that NN can easily use to learn non-linear functions. We used a very powerful embedding model called “FastText”^37^, which is trained on Wikipedia pages. FastText produces an embedding with a dimension size 300. The dimension size 300 is a good balance between vector quality and model complexity.

### Experiments and results

To evaluate the performance of the proposed CL-based phishing attack detection system, we performed experiments on VNN, TL, and CL models. For the CL experiments, we used two deep-learning techniques, LWF (a technique that enables an ML model to learn new tasks without forgetting previously learned knowledge) and EWC (a method that selectively reinforces the necessary weights of a NN to prevent catastrophic forgetting). In this section, we cover the details of the experiments and results. For each model, we trained six VNN models, each using a dataset, and then tested the trained model on all the datasets from 2018A to 2020B to compare their accuracy. The training, validation, and testing ratio is 80, 10, and 10 percent, respectively. Table 2 mentions the hyper-parameters used in all experiments. Moreover, the configuration we used for TL and CL experiments is the same as that of VNN experiments.

**Table 2:** Hyper-parameters used in experiments.

Approach	Learning Rate	Epochs	No of layers	Batch size
NN	0.001	100	4	50
TL	0.001	100	4	50
LWF	0.005	100	4	32
EWC	0.002	100	4	32

#### Experiments with VNN

**Figure 3:**
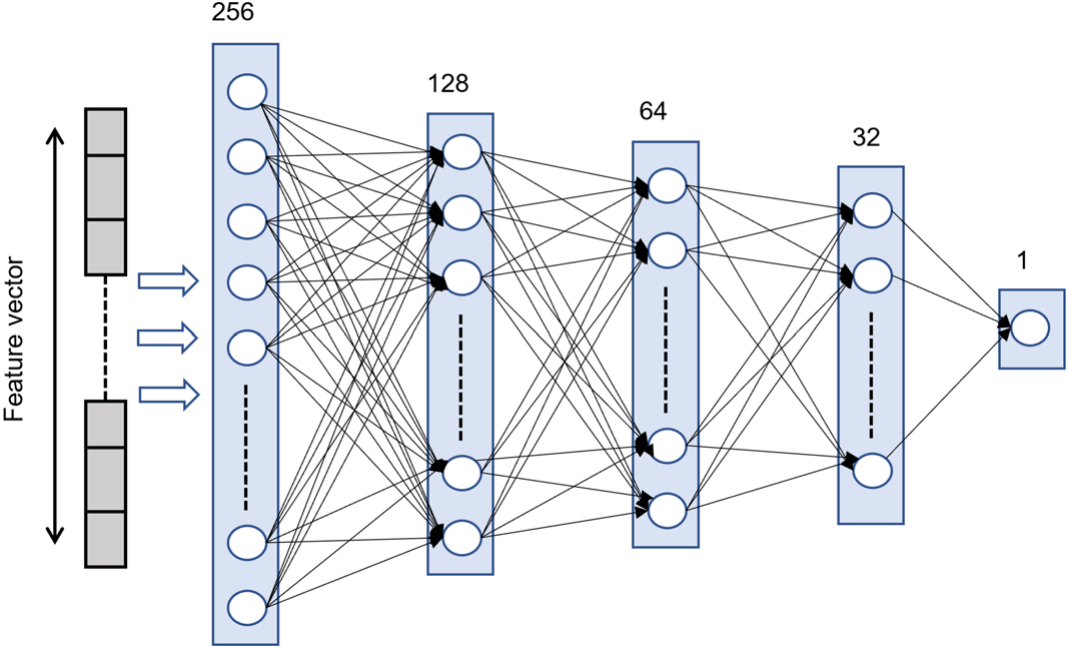
VNN architecture.

A VNN consists of an input layer, one or more hidden layers, and an output layer. Each layer comprises multiple neurons, where each neuron performs a weighted sum of its inputs, adds a bias term, and passes the result through a non-linear activation function such as ReLU, tanh, or sigmoid. Then, this result is passed to the subsequent layers. At the output layer, the error between the actual and predicted output is computed using a loss function, such as cross-entropy loss. This loss is minimized by using optimizers like SGD or Adam and backpropagated to the network by computing gradients at each layer.

Figure 3 shows the architecture of the VNN model that we have used in our experiments. An input layer that consists of a 300-dimensional feature vector. There are four hidden layers that have 256, 128, 64, and 32 neurons, respectively. Sigmoid activation and a dropout of 20% are used after each hidden layer. We have used the binary cross-entropy loss function with Adam optimizer for the binary classification task (phishing Vs. benign).

**Figure 4:**
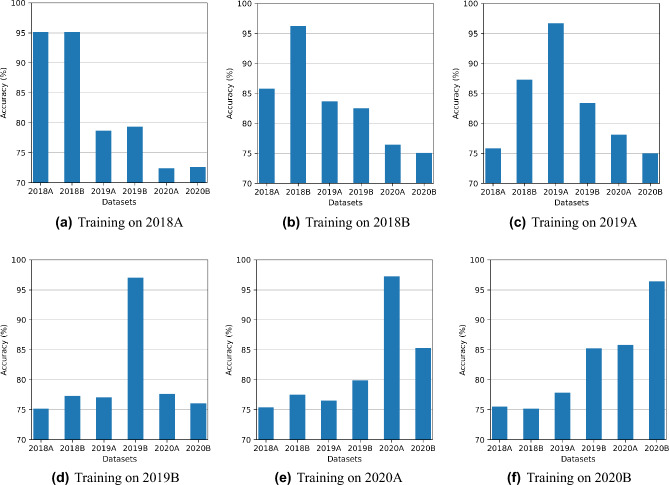
VNN only achieves high testing accuracy on the current dataset while not able to perform on remaining datasets.

Figure 4a–f shows a comparison of six experiments with VNN. Each experiment involves one training set at a time and is tested on all datasets. Experiments show that VNN gives high accuracy for the dataset used for training and lower accuracy on other datasets. It is clear from the results that VNN performs well on current tasks only and gradually drops accuracy on the previous tasks, i.e., Figure 4f shows VNN trained on 2020B achieves 96% on 2020B while the same model achieves less than 90% on previous datasets. In each experiment, the results achieved on the current dataset (that is used to train the model) are the best in terms of accuracy, so we use this as the benchmark to compare TL and CL accuracies.

#### Experiments with TL

**Figure 5:**
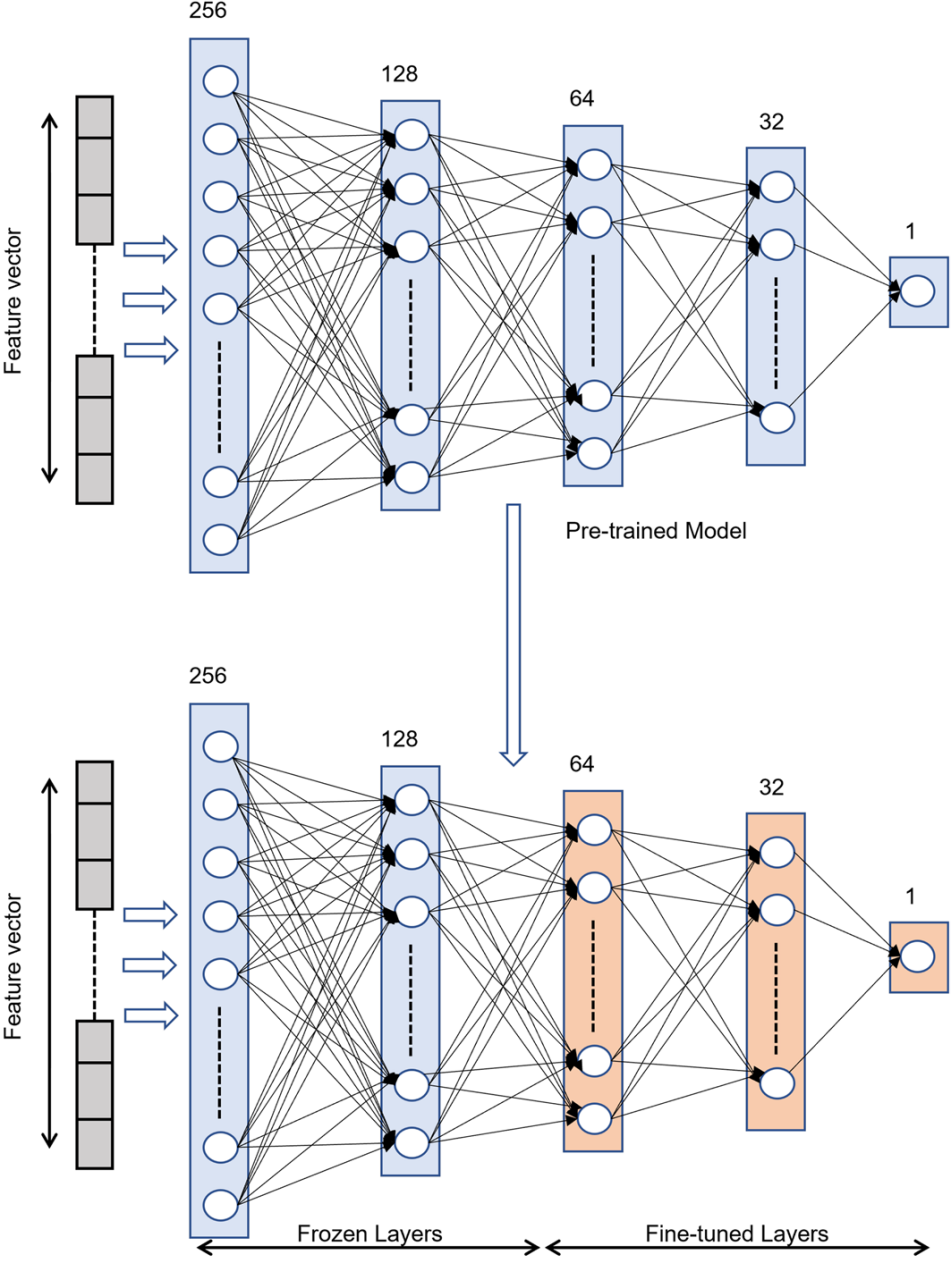
TL block diagram where red color shows fine-tuned layers.

TL is a well-known technique in deep learning where we fine-tune a pre-trained model (that is perfectly trained on a relevant problem) to a new task^28^. We retrain only the last few layers of the network and freeze the initial layers to use the generic features learned by these layers, as depicted in Fig. 5. This training regime reduces model convergence time, saves computation resources, and helps to learn with small datasets. However, in TL, the model works well only on new data and forgets the knowledge of old data. In our experiment, we fine-tuned the last two layers of the VNN architecture as shown in Fig. 5. The pre-trained VNN model is trained for the first chunk, 2018A, and tested on all other datasets. Then, the model is fine-tuned for the second 2018B dataset, and so on, fine-tuned with 2020B. It is observed that the more we retrain a model, the decrease in performance on the previous datasets is inevitable.

**Figure 6:**
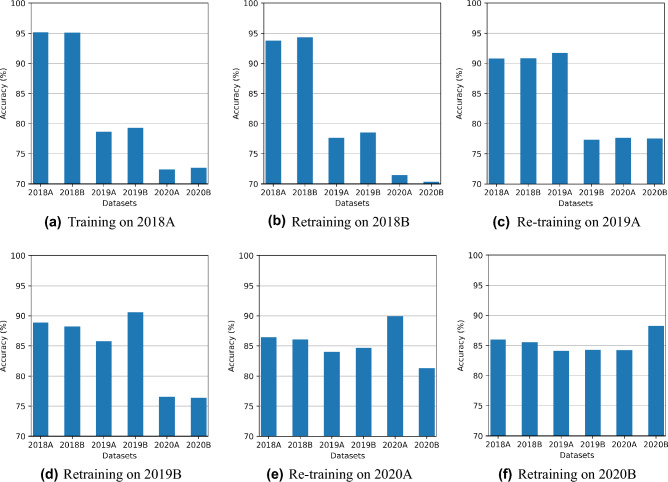
TL gradually decreases accuracy on previous datasets as the number of retraining tasks increases.

Figure 6 shows accuracy results using TL. TL makes some improvements in accuracy when a model is retrained on the following tasks. However, it is not the practical solution to this problem as it also deteriorates performance on the old tasks. Each experiment in Fig. 6a–f represents several retraining applied on the 2018A model, like experiment two on Fig. 6b represents when 2018A model is retrained with new dataset 2018B, it achieves 95% accuracy on 2018B and reduces accuracy on old task 2018A from 95% to 93%. Similarly, Fig. 6c shows that retraining the recently fine-tuned model for 2019A achieves 95% accuracy for it but reduces accuracy for both old datasets 2018A and 2018B. Finally, Fig. 6f shows accuracy for 2018A drops to 87% when retrained with five subsequent tasks while achieving high accuracy on 2020B. It is an inherent drawback with TL that it forgets previous tasks as moving forward.

#### Experiments with CL: learning without forgetting

CL is a training paradigm that allows the model to continuously adapt and learn new tasks without losing knowledge of the old ones, even without access to old data. Unlike TL, in which the model only works with new data, the CL technique is designed to work on both old and new data. Using the model trained on the old tasks, we apply CL incrementally to make it work on the dataset belonging to subsequent years. We used two state-of-the-art CL techniques known as learning without forgetting (LWF)^33^, discussed below, and elastic weight consolidation (EWC)^34^, discussed in the following subsection.

**Figure 7:**
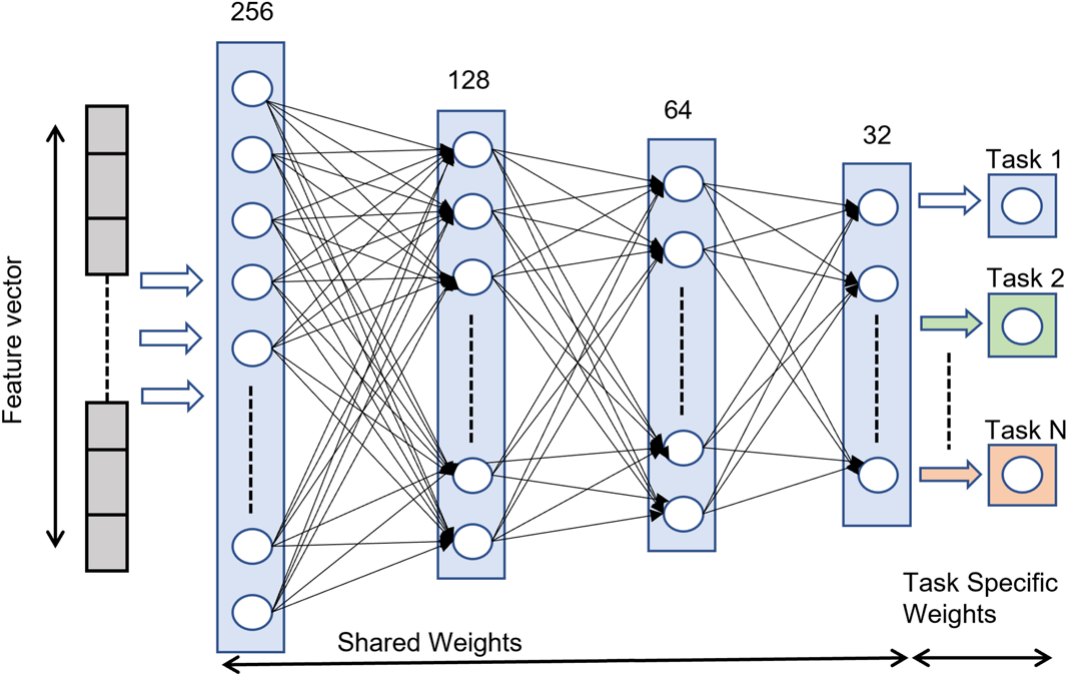
Learning without forgetting architecture.

In the LWF technique, we retrain the model on the new task and impose a penalty in the loss function that enforces it to maintain performance on the old tasks ( the task is as a set of input features and their corresponding targets used in the retraining of the model). Figure 7 shows two parts in the LWF model: (1) shared weights $\theta _s$ and (2) task-specific weights $\theta _o$. The shared weights contain the combined knowledge for all tasks, while task-specific weights are added on runtime as the number of tasks is increased in the future. LWF algorithm does not require data belonging to old tasks during CL. It is based on the intuition that by stabilizing the outputs from neurons belonging to old tasks on the new data, the model does not change the weights of the neurons essential for the old tasks.
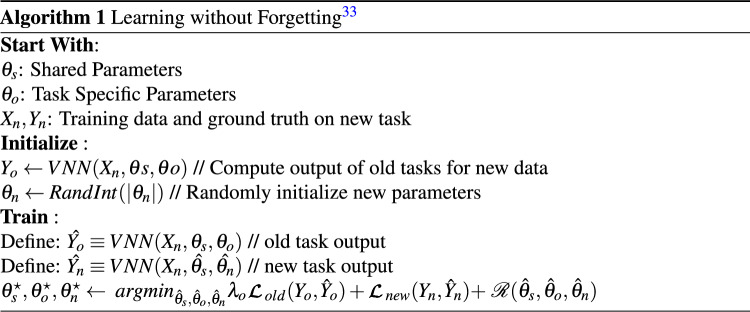


Algorithm 1 explains a step-by-step process to retrain the model with a new task. Our base VNN model, i.e., $VNN(\varvec{X_{n}, \theta {s}, \theta {o}} )$ is first trained on 20218A dataset, then a task-specific layer $\theta _n$ is added for a new task such as 2018B. Before any retraining, it records outputs $\it{Y}_o$ for the old task from the enhanced model. Then it trains the model to adjust the parameters to work well on old and new tasks using the data belonging to only new tasks. It performs two main computations during retraining, (a) it enforces the model to maintain the old task loss $\mathcal{L}_{old}$ as constant to make sure the model does not forget its current state for the old task, (b) reduces loss, i.e., $\mathcal{L}_{new}$, for new task heads (detection nodes) that are added to the decision layer with randomly initialized weights $\theta _n$. The network learns the new tasks and minimizes the loss for only new tasks by using regularization $\mathcal {R}$ with stochastic gradient descent^47^. A new task head can be added when we observe a performance decrease in the base model, as shown in Fig. 7. All task heads can be used in final decision-making (using majority voting or weightage-based decision), or only the latest task head can be used for the final decision. In our experiments, we trained a VNN model on 2018A, then retrained this model for the subsequent datasets using new task heads. It is observed that LWF sustains the detection performance as compared to TL. We used predictions from the latest task head to compute accuracy on each new task.

**Figure 8:**
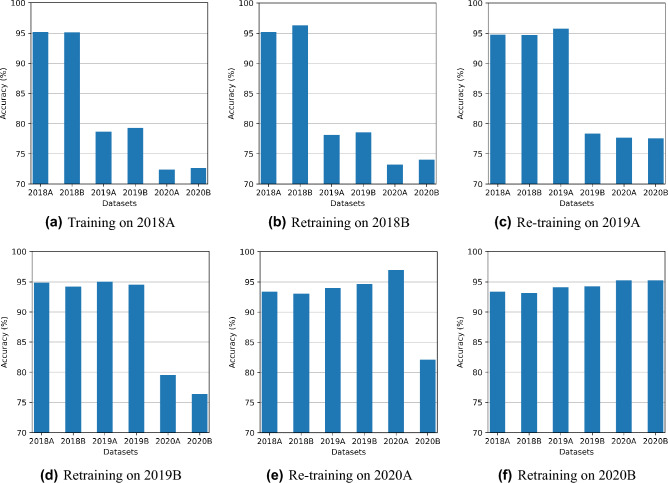
LWF sustains test accuracy on the current and previous datasets after retraining.

Figure 8a–f shows the results of CL with LWF. We observed that LWF accuracy increases for all datasets while maintaining performance on previous datasets. Similar to TL, each of the six experiments represents the number of retraining attempts that happened with the 2018A model, i.e., experiment 3 in Fig. 8c represents the results of the 2018A model after retraining on two new datasets. Figure 8f shows that LWF achieves 93% on the 2018A and achieves 95% on the last task after retraining five times. It shows that the CL method is quite effective in coping with forgetting problems on the old tasks while learning new tasks.

#### Experiments with CL: elastic weight consolidation

**Figure 9:**
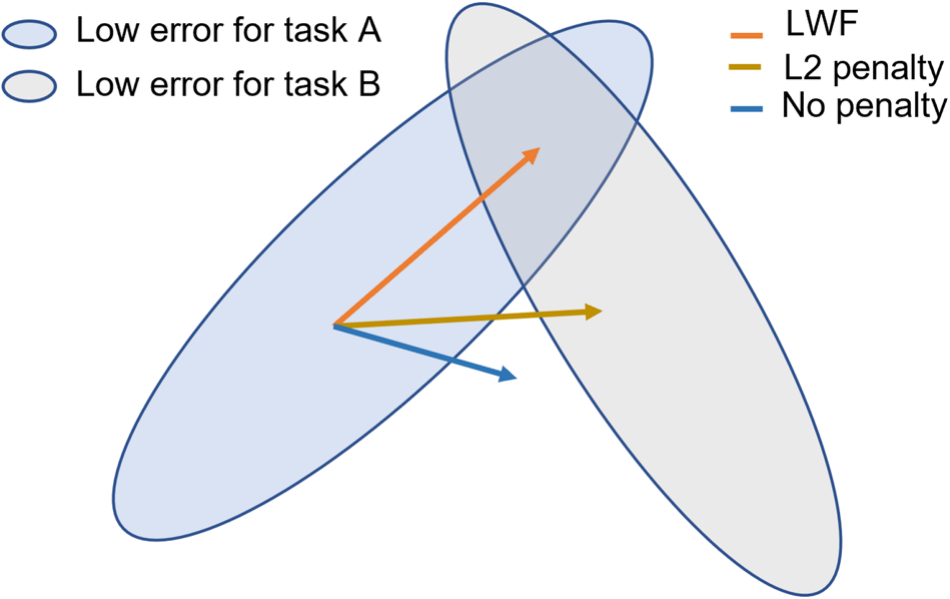
Elastic weight consolidation conceptual diagram.

Elastic Weight Consolidation (EWC) is another CL technique that is intuitive and effective in learning multiple tasks^34^. In EWC, we find the joint feature space between old and new tasks by adding a penalty in the loss function (Eq. (1)) of the VNN. Figure 9 shows two tasks, A and B, and we can find a common feature space for both tasks during the training. The penalty enforced during training of task B encourages the model to change only those weights that are not used or have less importance for task A. Consequently, the model learns a common feature space representing both tasks. EWC computes the weights’ importance for task A by computing the Fischer importance matrix *F*^34^ for training task B. The penalty in the loss function uses the Fischer matrix as shown in Eq. (1). The term $L_{B}(\theta )$ is the loss for task B, and $L(\theta )$ is the overall loss that is penalized with the F matrix. Overall loss decreases task B loss with a penalty to learn the common feature space between task A and B. It is observed that it is difficult to maintain high performance on old tasks as the number of tasks increases due to weights saturation.

1 $$\begin{aligned} L(\theta ) = L_{B}(\theta ) + \sum _{i} \frac{\lambda }{2}F_{i}(\theta _{i}- \theta _{A,i}^{*})^2 \end{aligned}$$

**Figure 10:**
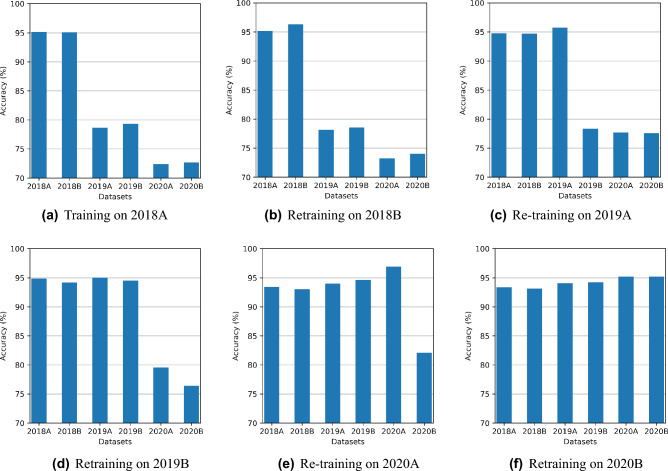
EWC also sustains test accuracy on old tasks but achieves slightly less test accuracy on current tasks than LWF.

Figure 10a–f shows the EWC method results. It shows that EWC also learns well on the current dataset and preserves the knowledge from the previous training, thus maintaining overall better performance accuracy for all tasks. For example, Fig. 10f shows that EWC achieves 93% accuracy on the 2018A dataset and achieves 95% on the 2020B dataset, which is quite close to LWF results.

From the above four experiments, we observe that VNN performs well only on the current task, TL gives some performance improvement, and CL-based methods give promising results for all tasks as shown in Figs. 4, 6, 8 and 10.

## Performance comparison of continual learning

This section describes the overall comparison of CL techniques with VNN and TL-based methods. CL techniques are observed to be resilient and reliable for achieving lifelong high-performance detection of phishing attacks.

### Accuracy comparison

**Figure 11:**
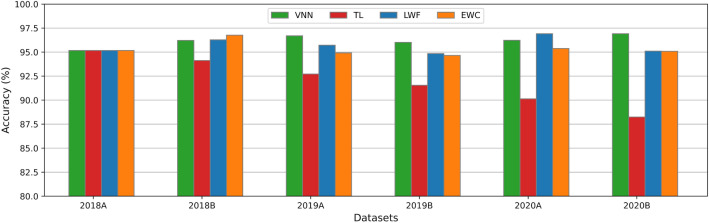
Comparison of TL and CL best accuracies with benchmark accuracy of VNN achieved on historical all datasets.

Figure 11 shows the performance comparison of best testing accuracies achieved with six datasets and all four techniques. VNN is trained separately for each dataset, while TL and CL models are retrained on each dataset. We see that VNN gives around 95% accuracy while there is a significant performance loss in TL, it loses its performance every time as the retraining tasks increase. While CL techniques outperformed as compared to TL approaches, and their accuracies are close to the VNN accuracy that is considered benchmark accuracy. Thus, CL-based algorithms give promising results for real-time model retraining.

### Confusion matrices

**Figure 12:**
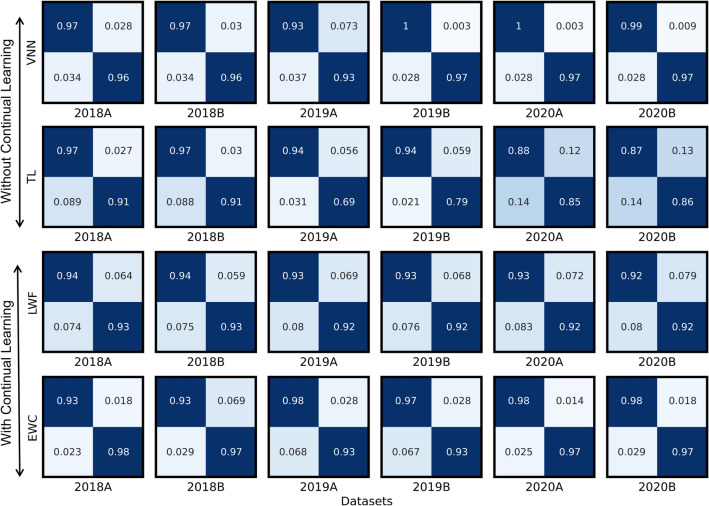
Confusion matrices comparison for best experiments from VNN, TL, and CL-based methods shows CL methods are close to VNN after retraining.

We compare confusion matrices for best performance from all experiments to evaluate VNN, TL, and CL techniques. A confusion matrix has four values: the first row represents true positive (TP) and false negative (FN) as the first and second columns, respectively. While the second row represents false positive (FP) and true negative (TN). In ML model evaluation, the TP value should be higher as it shows how well the model can identify malicious samples. Figure 12 shows confusion matrices for VNN, TL, and CL techniques with experiments on six datasets. It shows that VNN achieves a 97% TP score, the highest TP that is the best result without any retraining. At the same time, LWF gives 94%, which is the best result for all datasets with retraining. It can also be seen from Fig. 12 that VNN has a high true positive rate over the CL-based methods (LWF and EWC), which is computational vs. accuracy trade-off. Therefore, CL-based methods have achieved good accuracy over time, even with little retraining, while VNN requires training from scratch on each dataset. The FN score represents phishing pages that the model has miss-classified. We want the FN number to be as minimum as possible in the ML system. Our analysis shows that the false-negative score is overall lowest with the EWC method, as evident from Fig. 12.

## Discussions

This study investigates the issue of performance drop over time in ML models by retraining the trained model on only new data. To conduct our experiments, we consider two CL-based methods, i.e., EWC and LWF. These methods have relatively low computation and memory costs and require less training data and retraining time than other TL methods, e.g., DA and PNNs, etc. However, CL-based methods have several restrictions, e.g., (i) the size of learnable parameters increases as a new task is added for learning, (ii) catastrophic forgetting may happen after several training attempts by adding new data, and (iii) data bias, where CL model becomes biased for the specific task^48,49^.

We had seen in our experiments that when we subsequently trained the CL model on six datasets, it achieved satisfactory results comparable with VNN, even with little retraining on only new data. However, we observed a slight decrease in the true positive rate for CL-based methods because the CL model is trained on new data and may forget previously learned data (catastrophic forgetting). Therefore, it has reduced performance than VNN. Hence, it is a computational vs. accuracy trade-off. To the best of our knowledge, previous studies have used only one or more independent datasets to train and evaluate their ML-based methods for phishing detection. We did not use the datasets used in previous studies as they do not contain multiple years of data. We need multiple years of data to prove the idea of  CL in phishing detection. In this regard, we collected our own datasets for three consecutive years and present our results on existing ML-based phishing detection methods like vanilla neural network and  TL and compared them with  CL to show the life-long phishing detection performance.

## Conclusion and future work

In this study, we identified the performance drop of traditional ML models over time. To study the performance drop issue, we conducted several experiments under three different settings: a VNN model without retraining, a VNN model with TL, and a VNN model with CL. Our experiment results show that the VNN models have high detection accuracy when trained in a separate model every time for new datasets with different distributions. This requires training resources such as extensive training data and training time. We want to hold previous knowledge of phishing tactics and want to adapt it to new attacks. For this reason, we experimented with the TL-based model, and it deteriorated in accuracy over time when retrained with new data samples. CL algorithms have the most negligible effect on these changing data distributions due to an efficient retraining mechanism that adapts to new knowledge quickly while maintaining previously learned knowledge. Therefore, CL-based algorithms can be practically used as a first-stage phishing attack detection mechanism in a real-time environment with promising long-term results. CL-based algorithms have practical applications in reducing false positives, improving efficiency, and forming part of an overall phishing defense strategy.

In the future, further investigations can be performed to reduce catastrophic forgetting. As new phishing techniques are introduced, new tasks will be added, which will eventually increase the model’s complexity. This requires much retraining in hyperparameter optimization and finding the best possible set of parameters and model size, which can also be considered as future work. We also aim to explore the various embedding models, like BERT, etc., to extract more powerful features for phishing detection. We consider investigating other continual learning techniques, e.g., replay-based methods, task-specific learning, etc., in phishing detection as future work. Finally, we also consider the adversarial training of the CL-based models in the future.

## Data Availability

The dataset generated and analyzed during the current study of phishing attack detection is available in the Kaggle Datasets repository:https://www.kaggle.com/datasets/asifejazitu/phishing-dataset.
